
JOURNAL INFORMATION
==============================
NLM Title Abbreviation: Buffalo Med J
NLM Journal ID: 0027534

Buffalo Medical Journal

ISSN: 1040-3817
Publisher: Biomedical Journal Digitization Project

ARTICLE INFORMATION
==============================
PMCID: PMC8731437
PMID: 36886032
Article version: 1
Subjects: Item

Item

Print publication date: 1902 Sep
Volume: 42
Volume Series: New Ser
Issue: 2
First page: 152
Last page: 152
License: This work is free of known copyright restrictions. For more information, please see PMC Back Issue Digitization.
License URL: https://creativecommons.org/publicdomain/mark/1.0/

==============================

---

### ITEM.

Tarrant & Company, whose drug business has been carried on at 21 Jay Street, New York, since the disastrous explosion which destroyed their property on Warren Street two years ago, have sold their plant including good-will, trade marks, copyrights, merchandise, etc., to M. J. Breitenbach. The sale took place at public auction, June 23, 1902, and the price paid was $51,150. The business will be conducted by a new company of which Mr. E. G. Wells is president and Mr. T. F. Main, formerly the business head of Tarrant & Co., is the manager.

---

FOR SALE—A good location, for a physician near Buffalo, with an unopposed practice paying $1,800 to $2,000 a year, which will increase through industrious application. Property for sale or rent. Address: Buffalo Medical Journal.
